# Under which conditions do extreme events support a paradigm shift? Studying focusing events during two centuries of Swiss flood risk management

**DOI:** 10.1007/s10113-024-02316-2

**Published:** 2024-10-23

**Authors:** Anik Glaus, Alexandra Gavilano, Karin Ingold

**Affiliations:** 1 Institute of Political Science, University of Bern, Fabrikstrasse 8, CH-3012 Bern, Switzerland; 2 Oeschger Centre for Climate Change Research, University of Bern, Hochschulstrasse 4, CH-3012 Bern, Switzerland; 3 Eawag, Swiss Federal Institute for Aquatic Science and Technology, Überlandstrasse 133, CH-8600 Dübendorf, Switzerland

**Keywords:** Focusing events, Paradigm shift, Flood risk management, Policy process theories

## Abstract

Extreme weather events affect many areas around the world. How a country or region reacts to it can take many forms. In this article, we concentrate on policy responses, as typically found in laws, acts, or strategies. Recent research in climate change adaptation or environmental governance concluded that the degree of severity of extreme events is a crucial indicator that policy action should be taken. The event alone is a necessary, but insufficient condition for policies to be introduced. In this context, we ask: Which conditions must be at stake so that an extreme event is able to deploy its focal power and induce policy introduction or change? To answer this question, we studied more than two centuries of flood risk management in Switzerland. We relied on qualitative and quantitative data, as well as process tracing techniques, to relate event characteristics, media, political, and policy contexts to policy change in flood risk management. Results indicate that two conditions made floods turn into focusing events and support paradigm shift: high economic damage and a policy subsystem’s actor constellation favorable to change. We are convinced that our results are also replicable for other natural disasters and other countries than only Switzerland.

**Supplementary Information:**

The online version contains supplementary material available at 10.1007/s10113-024-02316-2.

## Introduction

Extreme weather events hit hard all over the world, particularly in Europe, during the last years and summers (CNN 2023; WMO 2021; IPCC 2021). While some countries could better cope with extremities, such as strong precipitation or heatwaves, others found it difficult to cope, which led to events with major damage in some regions (BBC Weather 2021; Gibbens 2021; Mathiesen and Cokelaere 2021; Patel-Carstaires 2021). The fact that the same extreme event leads to different outcomes is not just due to changing meteorological, physical, and socio-demographic circumstances in the diverse regions; over the last decades, different European countries have developed diverse climate adaptation strategies (Begum et al. 2007; Biesbroek et al. 2010; Bradford et al. 2012; Thaler and Hartmann 2016; Seebauer et al. 2023). Thus, extreme weather events in the past led to policy innovation and changes in some countries and regions, while not in others (see also Seebauer et al. 2023; Itsukushima et al. 2021). This key observation guides the research in this article, where we ask:

Under which conditions does an extreme event deploy its focal power and induce policy change?

To answer this question, we studied two centuries of Swiss flood risk management and investigated which extreme flood event was able to lead to policy change and which was not. Thereby, we focused on the characteristics of the event itself, as well as the media, political, and policy contexts present when the event happened. We relied on qualitative and quantitative data, and undertook process tracing in order to link event characteristics, media attention, and subsystem dynamics to policy change.

We followed the recent literature on extreme events and policymaking and tried to follow their claim that event characteristics and “policy” or “community” characteristics should be brought together more systematically to see when and whether an extreme event deploys its potential focal power and induces change (Birkland and Warnement 2016; Giordono et al. 2020; Klüver and Giger 2012). We triangulated the here-cited research on focusing events with different insights, learnings, and concepts borrowed from diverse policy process theories (Baumgartner and Jones 1993; Kingdon 1984; Sabatier and Jenkins-Smith 1993). In doing so, we were able to present a framework figure inspired by the various conceptual strands. To gain a more systematic insight into the relationship between events and policymaking, we undertook a longitudinal study.

Our results largely confirm what the literature says (Birkland and Warnement 2016; Giordono et al. 2020): characteristics of the event (such as its magnitude and damage) are necessary, but insufficient conditions. The event must emerge in a favorable “policy context,” with (dominant) advocacy coalitions in favor of or promoting change. The public and political context, measured through media and parliamentary attention in the aftermath of the event, plays less of a role. We are convinced that our approach does not only apply to natural but also to other extreme disasters and, therefore, also to the policy fields other than exclusively environmental ones.

## Theory

**Fig. 1:**
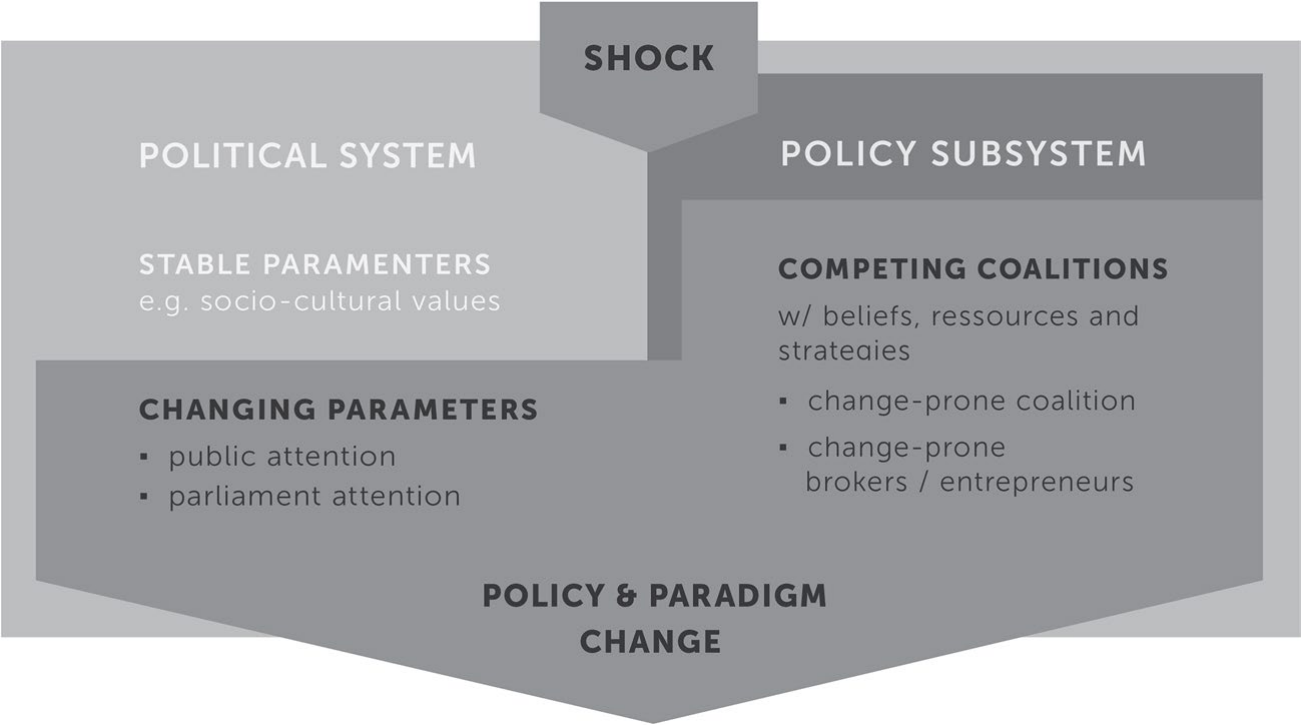
Adapted from Sabatier and Weible (2007), further inspired by Klüver and Giger (2012), Birkland (2006), and Birkland and Warnement (2016)

From the literature, we know that some shocks, such as accidents or natural disasters, lead to policy change, and some not. Sometimes, the very same event (such as the Paris agreement in climate politics, or the nuclear accident in Fukushima) deploys very different reactions (Klüver and Giger 2012; Peterson 2021; Wurzel et al. 2019). In that case, it cannot be the event’s characteristics that determine only whether a policy change happens or not. This is also in line with recent conceptual and empirical research on focusing events, where Birkland and Warnement (2016, p. 101), for instance, claimed that events and policymaking need to be linked more systematically: researchers need to know more about both the event’s features on one side and the policy process on the other side. This is also what we propose here. We outline a framework figure (see Fig. 1) that is inspired by both the most recent research on focusing events on one side (Birkland and Warnement 2016, 2017; Giordono et al. 2020) and by established policy process theories on the other side.

### Shocks, external, and internal events

Shocks are events coming from both inside or outside the policy community, elite, or subsystem. This is why we have drawn them on the edge of the political system in Fig. 1 (Birkland 1997; Sabatier and Weible 2007; Weible and Nohrstedt 2012). External events are typically natural disasters or accidents that happen outside of politics (broadly speaking), whereas internal events happen inside the political or policy (sub-)system and embrace a drastic change in the political regime or the dominant/governing coalition. All these shocks can challenge the existing sociopolitical order as well as political and public actors’ behavior, and hence, they may lead to drastic policy changes (Hall 1993; Sabatier 1988; Sabatier and Jenkins-Smith 1993).

### Policy subsystem and coalition dynamics

This box is also directly copied but simplified from the ACF (Sabatier and Weible 2007). A policy subsystem consists of a geographic scope, a topic or policy problem to solve, and the actors engaged in this topic or problem solving (Jenkins-Smith et al. 2018). Within a subsystem, actors informally coordinate actions with others who are like-minded and thereby form so-called advocacy coalitions to influence policy decisions, outputs, or impact (Sabatier and Weible 2007). Therefore, actors in the same coalition share beliefs, resources, and strategies.

### Political system

The policy subsystem is embedded in the larger political system that is usually characterized by stability. Stable parameters include the distribution of (natural) resources, sociocultural values, and macro-level institutions (Sabatier and Jenkins-Smith 1993). When an external shock arrives, there are some elements of the political system that are also prone to change. This typically includes public or media attention to an issue (Jones and Baumgartner 2005) or short-term constraints or opportunities (Weible et al. 2009).

### Policy and paradigm change

It is very rare to observe the creation of new policy outputs nowadays. This is why an important part of policy process research (Pierce et al. 2017) focuses on change rather than innovation. In general, policy change can happen in different ways, ranging from incremental shifts in previous policies to the implementation of new innovative policies (Bennett and Howlett 1992). Numerous theoretical frameworks and empirical applications exist that seek to define policy change and explain under which conditions political content, programs, and legal acts change (Roland 2004; Knill and Tosun 2012; Sabatier and Weible 2014). Seminal work by Hall (1993) “Paradigms, Social Learning, and the State,” is one of the most cited studies considering policy change, addressing long periods of political stability and shorter intervals of radical change (Baumgartner 2013). Hall (1993) described three types of policy change, increasing in their intensity from the first to the last: first-order change, i.e., incremental change to existing policies; second-order change, i.e., change in policy instruments; and third-order change, i.e., change in policy goals. The third and most profound level of Hall’s categories of change is understood as a paradigm shift. This extreme form of policy change concerns a shift in the ideas and discourses, i.e., the paradigm widely shared by an entire policy community, which is reflected in an ideological redesign of the community’s adopted policy goals and instruments (Cairney and Weible 2015; Daigneault 2014; Sabatier and Weible 2007). In other words, policy change is usually minimal and happens only incrementally to existing policies because the predominant paradigm supports the status quo. However, during some rare occasions, e.g., the occurrence of a focusing event, a paradigm shift happens and transforms policies themselves, resulting in a new equilibrium and a break from the past (Baumgartner 2013). As a paradigm shift can be so encompassing, in Fig. 1, we have drawn it at the edge between the sub- and broader political systems (also and potentially affecting other policy subsystems). The change we are interested in is abrupt but important: this is why we expect an interplay between the left and the right boxes of Fig. 1, and what Roland (2004) would describe as fast-moving (political) and slow-moving (ideological) changes.

### Linking shocks to change—the emergence of focusing events

Not all shocks lead to policy change. What Fig. 1 shows is that other parameters (changing, maybe sometimes also stable ones) and subsystem factors (such as resource distribution across competing coalitions) also need to be triggered by a shock to induce policy or paradigm change. Internal or external events become focusing events only when they activate policy reformulation dynamics and induce major policy change, which can ultimately lead to a paradigm shift in a certain policy subsystem. Or as Nohrstedt and Weible (2010) emphasized: the higher the policy and geographical proximity of a crisis or shock, the higher the propensity of change. In other words, chances for change are higher if a shock is very close, be it in terms of geographical distance, or between the nature of the shock and the needs in the subsystem. When shocks are in policy proximity, they can become focusing events that highlight policy deficiencies, the ineffectiveness of the current policy design or policy failures that can discredit the existing status quo (see Birkland 1998; Bergsma 2016; 2018). Policy failures provoke strong political and public pressure to which governments might respond by adapting previous policies or advancing new policies on the agenda (Walgrave and Varone 2008). Governments’ active search for improved or new policy solutions leads to an increased likelihood of policy change. Therefore, focusing events can be understood as triggers for policy change (Baumgartner and Jones 1993): they are able to open a policy window or “window of opportunity” (Birkland and DeYoung 2012; Kingdon 1984), which provides the possibility to implement new policies and foster policy learning. Thus, focusing events activate a process that brings decision-makers to move away from the deeply rooted status quo toward the idea that policy change is necessary (Birkland 2004).

Various policy process theories have paradigm shift as the focus of their attention, and they consider external or internal events to be a driving factor for major policy change. To identify conditions turning an extreme event into a focusing event, we considered elements of the punctuated equilibrium framework (PET) (Baumgartner and Jones 1993) and the advocacy coalition framework (Sabatier and Jenkins-Smith 1993). Based on these frameworks, we analyzed and discussed in the following sections three sets of natural and sociopolitical conditions that potentially lead external events to deploy their focal power and induce major policy change resulting in a paradigm shift: the shocks’ magnitude, triggered changing parameters (e.g., media attention), and the shocks’ potential impact on the policy subsystems (e.g., competing coalitions and single actors).

### Shocks and their magnitudes

Following Birkland’s definition, one decisive characteristic of focusing events is the *magnitude* of the harm caused (Birkland 1997). A focusing event’s focal power is directly linked to its magnitude, which varies in intensity; some large-scale events affect a whole society, while others are more moderate and have local effects (Birkland 1997; Lindholm 2016; Lindholm et al. 2015). As Lindholm (2016) argued, discussing the magnitude of a focusing event is important when considering the politicization of focusing events, i.e., the higher the magnitude of a focusing event, the higher its focal power and its impact on policies.

To assess the magnitude of an extreme event, and thereby hypothesize about its policy impact, researchers have focused on the extent of response requirements (Birkland 2006; Quarantelli 1987), the number of deceased (Oh and Oetzel 2011), infrastructure or economic loss (Yan and Bissell 2018), or geographic and cultural proximity (Lindholm 2016). Regarding the response requirements, we can differentiate responses on a scale, from an accident that only requires the activation of established response organizations, such as an emergency service, to an emergency requiring the mission of a latent response organization, such as an army service, to a catastrophe when the introduction of new organizations can be observed (Lindholm 2016). Regarding the number of deceased and economic losses, the higher this number, the more focal and powerful an extreme event becomes. The more an event has a negative effect on a large number of people, the more an event generates high attention in society, and the greater the social pressure on politics to react (Alexandrova 2015). However, a natural disaster in a regional context with a few deaths and moderate economic losses, although devastating for this region, does not have the same focal power as a natural disaster on a global or national scale, with a potential of hundreds or thousands of deaths and high economic losses. In relation to proximity, the importance and magnitude of an extreme event increases when people are directly affected, either because the event takes place in their immediate geographic proximity or because they feel a common sociocultural or historical proximity (Lindholm 2016). All of the abovementioned factors point to the fact that the objective or measurable magnitude of an extreme event decisively wields focal power on politics. Therefore, our first hypothesis is as follows:

- *H1:The magnitude of a shock is one necessary but insufficient condition for an event to deploy focal power and induce paradigm shift.*

### Shocks and their public attention

The punctuated equilibrium framework developed by Baumgartner and Jones (1993) argued that a paradigm shift requires a considerable degree of public attention to shift decision-makers’ agenda priorities, e.g., through media coverage, social mobilization, or parliamentary activity. External events and their high levels of pressure can act as important triggers or catalysts to enhance public attention (see Jones and Baumgartner 2005). More concretely, the event can impact both the competition over which problems are most important and the competition over which causes and solutions surround any one problem (Birkland and Lawrence 2009). Policy issues induced by focusing events benefit from extensive media coverage and high parliamentary attention and have the potential to change the image of how a problem is perceived and, consequently, how a policy is framed (Baumgartner and Jones 1993). An extreme event becomes focal the more it expands via media or political discussion to a wider public, and the more it reaches a high number of interested citizens (Birkland 1997).

Thus, to measure public attention and agenda activity (see again Birkland and DeYoung 2012), our second set of hypotheses reads as follows:

- *H2a: After a shock, high media attention is an important condition for an event to deploy focal power and induce paradigm shift.*
- *H2b: After a shock, high parliamentary attention is an important condition for an event to deploy focal power and induce paradigm shift.*

### Shocks and their impact on policy subsystems

The ACF by Sabatier and Jenkins-Smith (1993) argued that minor policy change concerning secondary beliefs (e.g., policy preferences) can be the result of learning or brokerage within a subsystem dominated by different competing coalitions, whereas major and thus fundamental policy change concerning policy core beliefs (e.g., fundamental values) is due to a shock from outside the political system. In other words, paradigm shift is most often a consequence of external shocks. Both frameworks, the ACF and PET, focus on the “human cognitive and emotional side of political decision making” (Jones and Baumgartner 2012, p. 4); however, PET relies on the allocation of decision-makers’ scarce attention, while the ACF is based on subsystems’ belief structures.

Following Sabatier and Jenkins-Smith (1993), shocks can have two important effects on a policy subsystem. First, they have the potential to redistribute critical resources (e.g., public support, financial means) that can change a subsystem’s power structure and, thus, replace a dominant advocacy coalition with another competitive (minority) coalition in the subsystem. Second, shocks may put into question a dominant advocacy coalition’s core beliefs, because such an event identifies policy failure and increases actors’ doubts about the effectiveness of the dominant coalition’s policy. Simultaneously, shocks may confirm a minority coalition’s core beliefs that may consequently become more active in the subsystem. Both effects influence actors and their decision-making within the subsystem, which in turn may result in major policy change (Sabatier and Weible 2007).

- *H3a: A shock absorbed by a subsystem including one change-prone dominant or change-prone (and reinforced) minority coalition is an important condition for an event to deploy focal power and induce paradigm shift.*

In the last scenario, in which coalitions in a subsystem compete only moderately (Ingold and Varone 2012), some single key actors might identify shocks as a chance or “window of opportunity” (Kingdon 1984), to enhance policy learning in the subsystem and, thus, induce policy change. Those key actors, following the ACF, are said to act as *policy brokers* who seek policy compromise and subsystem stability; they act in a belief-neutral way through across-coalition action (Ingold 2011; Ingold and Varone 2012; Sabatier and Jenkins-Smith 1993). In contrast, Kingdon (1984) (see also Birkland 1997; Zahariadis 2007) identified such key actors in situations of policy change rather acting in their own interests, seeking to translate their own ideas, discourses, and beliefs into policies. Those *policy entrepreneurs* exploit the “window of opportunity” to their own benefit and, unlike policy brokers, do not seek policy compromise, but bring innovation to the subsystem, and, thus, potentially induce policy change. These insights led us to our last hypothesis:

- *H3b:A shock absorbed by a subsystem including change-prone policy brokers or entrepreneurs is an important condition for an event to deploy focal power and induce paradigm shift.*

In sum, we want to study different conditions under which a shock develops its focal power and becomes a focusing event for paradigm shift. So we conceive paradigm shift as a rapid but fundamental change in public policymaking and expect, similar to Roland (2004), that this change is induced by an interplay of short-term political and long-term, more ideological alterations (see left and right boxes in Fig. 1). More concretely, we expect the magnitude of a shock having an impact on its capacity to change policies (see Nohrstedt et al. 2021). But a shock of high magnitude is only a necessary but not sufficient condition for policy change. We further hypothesize that a major shock induces public and parliamentary attention, so two additional conditions for an event to deploy its focal power. On the contrary, we assume that a change-prone environment (subsystem with change-prone coalitions or actors) needs to be present already at the moment when the major shock arrives. In what follows, we want to disentangle the sequence of conditions leading to paradigm shift.

## Case

We analyzed the highly relevant case of flood events and the subsystem of flood risk management in Switzerland. Floods are typical extreme events with potential focal attributes (see Birkland 1997). Flood events and the subsystem of flood risk management in Switzerland prove to be an ideal case to investigate the conditions under which extreme events have the capacity to induce a paradigm shift for several reasons. First, severe flooding has often occurred in Switzerland in the past, which allows us to study a series of heavy flood events over two centuries. Second, Swiss flood events and their impacts are well documented and have been studied over the last two centuries (Löschner and Nordbeck 2020; Metz et al. 2020; Schmocker-Fackel and Naef 2010; Weingartner et al. 2003). This enabled us to compare several flood events over a long period. Third, by recovering from and preventing further flooding, Switzerland implemented a wide range of flood-related policy programs that made it possible to study paradigm shifts.

## Methodology

### Methodical approach to testing hypotheses

To test our hypotheses, we proceeded in three steps. In the first step, we identified paradigm shifts in two centuries of Swiss flood risk management. Therefore, we systematically reviewed all regulations related to flood risk management between 1848 and 2020, including the introduction or revision of constitutional articles, acts, and ordinances, as well as the establishment of strategies and platforms. The investigation of such a long time period ensures that broad trends in policy making, i.e., flood paradigms and major policy changes in flood risk management can be detected (Jones 1994). In addition to identifying major policy changes in regulations, we systematically coded the dominant flood regime. This signifies that we particularly analyzed the prevailing paradigms in Swiss flood risk management, which related to the basic idea and ideological tone and the way in which flood policies and instruments are framed and designed (see also Hall 1993). Once a new ideological framing is visible in the relevant regulations, we coded it as a paradigm shift.

In the second step, we identified hydrologically defined major flood events in Switzerland. We initiated our historical flood analysis in the mid-nineteenth century with the beginning of hydrometeorological observations being recorded. Based on the floods’ return period, hazard level, and runoff capacity in the respective hydrological catchment areas, we categorized flood events according to their magnitude (Flügel 2000). Then, we selected flood events representing a high or very high risk (levels 4 or 5 of 5) (see article 10 in the Swiss Alerting and Security Radio Ordinance) (FEDLEX 2019) and showed, on average, a return period equal to or higher than every 30–100 years (HQ30–HQ100). Further, we drew on two additional criteria following Pfister (2009), of which at least one needed to be fulfilled: either a flood event is of national significance and shows a certain geographical outreach, i.e., affects at least five Swiss cantons simultaneously, and/or a flood event causes damage of over CHF 500 million (CHF reference year of 2009).

In the third step, we linked paradigm shifts to major flood events to study conditions that turn floods into focusing events. Therefore, we first analyzed whether certain major policy changes and major flood events can be identified as occurring around the same time period. When major policy change can be potentially related back to a major flood event before or during the same time period, this flood event deploys focal power and induces paradigm shift. To subsequently identify the conditions turning a flood into a focusing event, we compared flood-inducing paradigm shifts to those that do not. The conditions we analyzed included a flood event’s magnitude, media and parliamentary attention triggered by a flood event, and subsystem properties in the field of flood risk management.

### Variables, data, and methods

To operationalize our variables, we used a combination of qualitative and quantitative data retrieved from different databases. We analyzed our data by applying a mixed-methods approach.

The variable *paradigm shift* is based on a number of constitutional articles, acts, ordinances, and strategies related to the prevention of flood events (see Table 2 in the supplementary online information for the full list of considered regulations) as well as on secondary literature, such as reports, documentations, and dissertation theses on Swiss flood risk management (see Burger 2008; Summermatter 2012; Zaugg 2006). We conducted a document analysis and focused on the introduction or revision of regulations and their content. Once a new framing of Swiss flood risk management is activated in these regulations, we cross-checked with the secondary literature for major historical policy changes and validate eventual paradigm shifts.

For *major flood events*, we relied on the Swiss flood and landslide damage database, categorizing, and evaluating each flood event in Switzerland, provided by the Swiss Federal Institute for Forest, Snow, and Landscape Research (WSL). In addition, we used statistics originating from official databases provided by the Swiss federal administration (see Bezzola and Hegg 2007; Bezzola and Ruf 2009; FOEN 2012a, 2012b; FOWG 2000, 2002). To validate this data, we drew on secondary literature on major Swiss flood events (Pfister 1999, 2002; Röthlisberger 1991; Summermatter 2012). Thus, we expanded the analysis of quantitative data with findings from a document analysis of the secondary literature.

To link paradigm shifts to major flood events and, thus, to evaluate whether flood events deploy their focal power, we applied the method of process tracing (George and Bennett 2005; for an application, see Walgrave and Varone 2008). Process tracing is applied prominently in within-case analysis. Accordingly, we treated each paradigm shift and its potential link to a major flood event as one case. In doing so, we followed two important steps typically applied in process tracing (Collier 2011): first, we systematically described all variables, i.e., each paradigm shift and each flood event, and second, we identified sequences to link these variables to each other, i.e., one particular paradigm shift to one potential focusing event. We based this process tracing method on secondary literature (Burger 2008; Summermatter 2012).

To identify conditions that turn floods into focusing events, we analyzed four different conditions. The first condition, *flood events’ magnitude*, was measured through the number of fatalities a flood event causes, the geographical outreach it had—i.e., the number of affected Swiss cantons—and the economic damage in Swiss francs it raised (according to Röthlisberger 1991). Data and statistics for the evaluation of flood events’ magnitude originate from the aforementioned databases provided by the WSL and the Swiss federal administration. For economic damage, which depends upon socioeconomic factors such as death, inflation, or currency value, we further considered figures retreated by historians to adjust values over a large time period (Pfister 1999, 2002).

To study the second condition, *media attention after flood events*, we considered media coverage (number of articles) during 1 year after the flood event occurred in four leading newspapers in the German-speaking part of Switzerland: Neue Zürcher Zeitung (NZZ), Tagesanzeiger (TA), Neue Luzerner Zeitung, and Blick. The first (an elite newspaper) and the last (a boulevard-type newspaper) have a national outreach, whereas the other two (high-quality newspapers) have a stronger regional focus. For the period between 1910 and 2005, we relied on a study published by Zemp (2015) and applied the same method of media analysis for the period until 2020.

For *parliamentary attention after flood events*, we analyze the number and issues of parliamentary interventions (including all different types, e.g., motions, postulates, interpellations) during two parliamentary sessions following each major flood event (time period of 4–6 months; Table 3 in the supplementary online information). Concretely, if a parliamentary intervention relates to the issue of flood risk management, natural hazards in general, or renaturation as a prevention of flooding, we included it in the analysis. As a database, we used the parliamentary archive of the Swiss Confederation (Curia Vista).

The *properties of subsystems absorbing flood events* is an antecedent condition (see Table 1). This means, that we expect the subsystem properties having evolved already before the event occurring to create a “change-prone” environment. In line with Roland (2004), but also Nohrstedt and Weible (2010), see also Nohrstedt et al. 2021), we thus expect that paradigm shift and thus rapid but fundamental changes are only possible when abrupt triggers such as flood events interact with slow changes at the cultural, normative, or ideological level. To grasp this ideological dimension, we study the national subsystem of Swiss flood risk management and elite actors therein. More concretely, we are interested in the identification of two subsystem elements (see also Fig. 1). On the one side, we study the so-called advocacy coalitions, thus groups of like-minded actors. Typically, a paradigm shift is only possible if a change-prone coalition is also supporting this fundamental change. On the other side, we pay also attention to single actors and their roles in the subsystem after major flood events: so-called policy brokers and entrepreneurs are those actors advocating for policy compromise among opposing coalitions or policy change and might thus play a particular role. In sum, we were particularly interested in whether, when, and how actors and actor groups form coalitions and how these coalitions influence the design of flood policies. We more concretely hypothesized that *change-prone advocacy coalitions* or *change-prone brokers or entrepreneurs* is an important condition to turn a flood into a focusing event. To analyze subsystem properties, we relied on secondary literature on Swiss flood risk management (Burger 2008; Müller 2004; Pfister 1999, 2002; Summermatter 2012), as well as studies of the overarching water policy subsystem (Bolognesi and Nahrath 2020; Brandenberger et al. 2021; Kissling-Näf and Kuks 2004; Rohr et al. 2018; Varone et al. 2002).

**Table 1:** Key variables and conditions and their expected sequence

Variable and expected sequence	Operationalization	Data
Dependent variable		
Paradigm shift in Swiss flood risk management	Introduction of new regulatory frameworks, laws and ordinances, or substantial amendment of existing ones (e.g., new constitutional article)	Document analysis of Swiss Constitution, legal texts, see Table 2 in the Appendix Secondary literature
Antecedent variables		
Change-prone advocacy coalitions	Identification of groups of like-minded actors in favor of the regime change	Secondary literature on Swiss flood risk management (Burger 2008; Müller 2004; Pfister 1999, 2002; Summermatter 2012), and the overarching water policy subsystem (Bolognesi and Nahrath 2020; Brandenberger et al. 2021; Kissling-Näf and Kuks 2004; Rohr et al. 2018; Varone et al. 2002)
Change-prone individual actors	Identification of individual actors in favor of the regime change, and having the position to advocate compromise (brokers) and change/innovation (entrepreneurs)	
Independent variables		
Major flood events	Magnitude of flood event related to fatalities, damage costs, and affected geographical area	See Table 2 Swiss Flood and Landslide database Secondary literature
Media attention after flood events	Issue attention (number of articles reporting about the occurred flood until one year after the event)	Newspaper analysis (four leading Swiss newspapers) Secondary literature
Parliamentary attention after flood events	Interventions by parliamentarians the two sessions after the event	Text and document analysis

**Table 2:** Major flood events between 1848 and 2020 with their number of fatalities, cantons affected, and the economic damage sum

Year	Dates^a^	Fatalities^b^	Cantons affected^b^	Economic damage^c^ (million CHF)
**1868**	27.09.–04.10	50	5	1436
1876	10.–15.06	-^d^	13	826
1910	14.–15.06	27	21	584
1978	06.–07.08	9	13	930
**1987**	18.07.; 24.–25.08	8	5	1270
1993	24.09.; 12.–13.10	3	2	735
1999	11.–15.05.; 20.–25.05	4	8	552
2000	14.-15.10	16	4	560
**2005**	20.–23.08	6	16	2800
2007	08.–09.08	1	22	375
2011	10.–11.10	0	6	62
2013	01.–02.05.; 31.05.–02.06	2	7	79
2014	22.–24.07.; 10.–11.08	0	6	27

^a^In some years, more than one flood occurred. We considered a maximum of one flood event per year because over our large investigated period of two centuries, two, or more subsequent floods in a short period can be understood as one (reinforced) flood event

^b^Data on flood fatalities and affected cantons is based on flood event analysis by the Federal Office for the Environment (Bezzola and Hegg 2007; Bezzola and Ruf 2009; FOEN 2015; FOWG 2000, 2002) and on secondary literature (Petrascheck 1989; Pfister 2002; Röthlisberger 1991; Summermatter 2012)

^c^For the floods in 1868, 1876, and 1910, the economic damage data are based on Pfister (1999, 2002) and Röthlisberger (1991). To compare historically documented economic damage with today’s situation, Pfister (2002, p. 216) related a flood’s economic damage to contemporary wage levels. Based on the average increase in nominal wages in the construction industry since a flood’s occurrence, the economic damage at that time can be estimated according to today’s situation. For instance, hourly wages in the construction industry increased by 105 times between 1868 and 2000, i.e., the 1868 flood’s damage of original value CHF 13.74 million translates to a current damage of CHF 1436 million (original value in 1876: CHF 14 million; 1910: CHF 16 million). For the more recent data on economic damage (1978–2014), we relied on the Swiss flood and landslide damage database by the Swiss Federal Institute for Forest, Snow, and Landscape Research WSL (Hilker et al. 2009). The economic damage data were adjusted to inflation in 2005

^d^No data available for flood fatalities in 1876

## Results

### Paradigm shifts in Swiss flood risk management

**Fig. 2:**
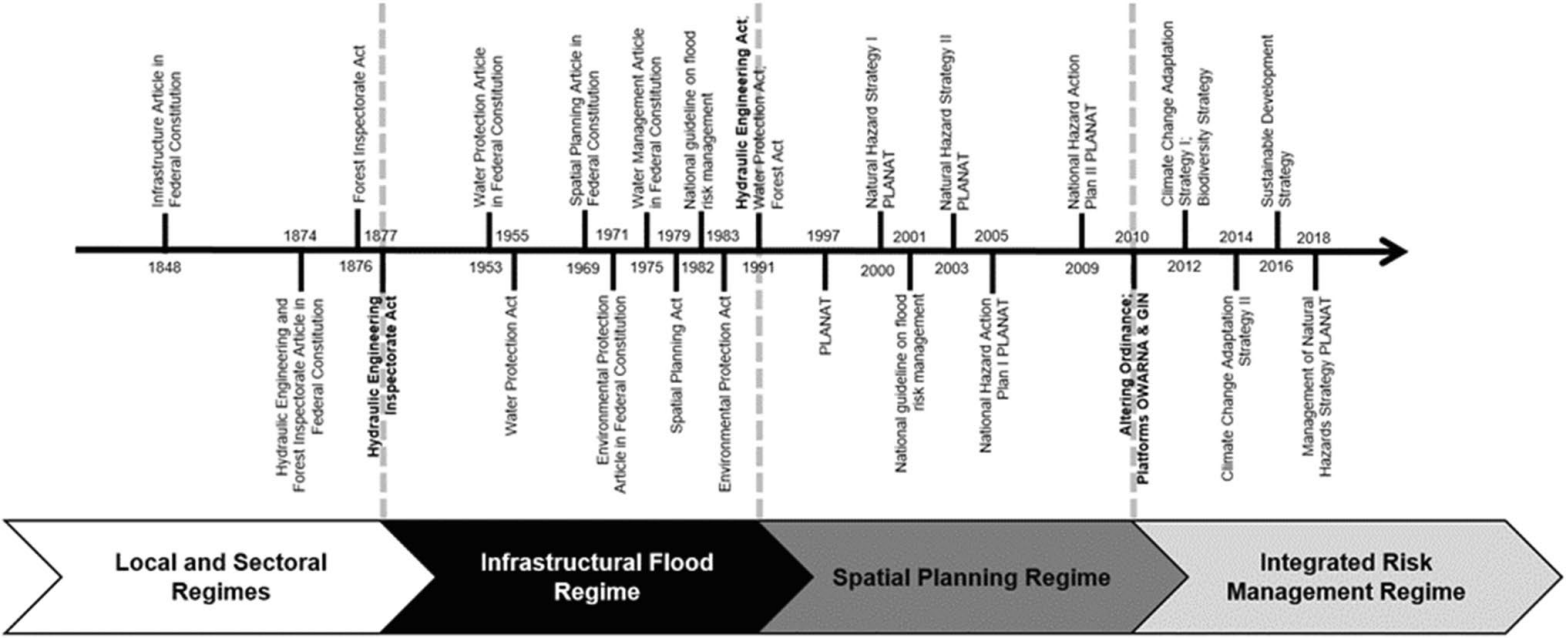
Regulations, regimes, and paradigm shifts in Swiss flood risk management

We observed three important paradigm shifts in Swiss flood risk management (see Fig. 2).

The first paradigm, until the beginning of the nineteenth century, was primarily characterized by local flood regimes organized by affected municipalities (Zaugg 2006). These local flood regimes were soon replaced by the first major river corrections of the Linth in 1807 and the Jura waters, Rhine, and Rhone after 1860. An infrastructure article in the Swiss Constitution of 1848 further institutionalized this focus on major river courses. In 1874, the constitutional revision further transferred hydraulic engineering competences from the local and regional levels to the national level. The paradigm shift toward the so-called infrastructure regime (see Fig. 2) was then realized with the introduction of the Hydraulic Engineering Inspectorate Act in 1877 (and, to a lesser extent, the Forest Inspectorate Act in 1876). It pushed local and sectoral flood regimes to develop into one dominant national infrastructure regime, considering the same technical standards for whole river catchments, including tributaries and forests (Rohr et al. 2018).

The infrastructural regime dominated Swiss flood risk management for more than a century. The emerging debate on environmental protection from the 1950s onwards and the introduction of articles on water protection (1953), spatial planning (1969), environmental protection (1971), and water management (1975) into the Federal Constitution, however, changed the exclusive focus of flood risk management on infrastructure (Summermatter 2012). A new trend of bottom-up environmental activism developed around the 1970s, which resulted in the Spatial Planning Act being installed in 1979. These developments also impacted Swiss flood risk management, where spatial planning approaches gained importance. Finally, in 1991, with the introduction of the Sustainable Hydraulic Engineering Act, a new regime was born: the spatial planning regime.

But a quick transformation happened in the late 1990s and was reflected, on the one hand, in the establishment of the extra-parliamentary National Platform for Natural Hazards (PLANAT) in 1997 and, on the other hand, in the introduction of the new national guideline on flood risk management in 2001, promoting an integrative approach by combining the goals of flood risk management, water protection, and water use. In subsequent years, several legal revisions related to flood risk management integrated sustainability principles, including a balance of technical, environmental, socioeconomic, and cultural aspects (Zaugg 2006). Furthermore, PLANAT designed a natural hazard strategy between 2000 and 2003 and an action plan between 2005 and 2009, both following an interlinked and cross-sectoral approach. Other sectors also started to address flood risks and watercourse regulations explicitly (e.g., water protection, forestry). This trend established a new paradigm in Swiss flood risk management, leading to a shift toward the integrated risk management regime.

### Major flood events and their focal power

We identified 13 major flood events in Switzerland between 1848 and 2020 (see Table 2 in supplementary online information), of which three turned out to be focusing events.

The 1868 flood was one of the largest and most significant in Swiss flood history. It affected five cantons simultaneously, caused 50 fatalities, and resulted in economic damage amounting to over CHF 1.4 billion. Public attention was notable, including ample nationwide and international solidarity, demonstrated by immediate repair work and fundraising campaigns (Summermatter 2012). According to several different sources, the 1868 flood deployed focal power and contributed to the emergence of an infrastructural regime (Burger 2008; Petrascheck 1989; Summermatter 2012). Therefore, we classified this flood as a focusing event.

The flood in 1876 concerned half of the Swiss cantons and caused economic damage of more than CHF 800 million (see Table 1 in supplementary online information). However, political reactions were cautious because demands for flood risk management were satisfied with the recent legal developments in 1874 and 1877 (Summermatter 2012). Similarly, the flood in 1910 influenced nearly all Swiss cantons, caused 27 fatalities, and totaled in economic damage of CHF 580 million. Nevertheless, legal revisions were only minor and concerned adjustments to existing practices (see Burger 2008).

The next two major floods occurred more than half a century later during the emerging environmental debate. The flood in 1978 had large outreach (13 affected cantons), caused 9 fatalities, and economic damage added up to CHF 930 million. This flood strengthened the idea of spatial planning elements in flood risk management, in particular the designation of risk zones, but it failed to be considered in the 1979 Spatial Planning Act (Summermatter 2012). In contrast, the flood in 1987 with more limited outreach (5 affected cantons) but huge economic damage (CHF 1.3 billion) is comparable to the 1868 flood. This flood encouraged intensive political debates about increasing the flood risk management budget, strengthening ecological elements in the law, and harmonizing the legal texts of hydraulic engineering, water protection, fisheries, and forestry. These suggestions were integrated into the new Hydraulic Engineering Act adopted in 1991, which prioritized spatial planning over technical measures (Rohr et al. 2018). Consequently, we identify the 1987 flood as a focusing event, while the 1978 flood cannot be classified as such.

Within the next decade, three floods in 1993, 1999, and 2000 occurred and caused similar economic damage. While the floods in 1993 and 2000 affected a few southern cantons, the flood in 1999 concerned 8 northern cantons. Despite decreasing numbers of flood victims (Pfister 2009), the 2000 flood caused 16 fatalities, most of which occurred in one village (Summermatter 2012). The three floods illustrated that intensive land use and limited space for watercourses are hazardous, and a more systematic and integrative coordination of natural hazard prevention is necessary. They may have had an impact on the establishment of PLANAT in 1997, as well as on the introduction of new guidelines on flood risk management in 2001 (Rohr et al. 2018). However, the prioritization of watercourses’ space requirements has been decided before. Thus, we cannot categorize the floods in 1993, 1999, and 2000 as focusing events.

Beyond the 1868 flood, the flood in 2005 was the largest and most disastrous in recent Swiss flood history. The 2005 flood affected the majority of the Swiss cantons, caused 6 fatalities, and resulted in economic damage of CHF 2.8 billion. An intensive political debate on the causes, effects, and lessons learned arouse, and the recovery process led to a number of developments that were strongly influenced by integrative, cross-sectoral, and multi-level elements, such as broad coordination and information platforms (“Optimierung der Warnung und Alarmierung” [Optimization of warning and alerting] (OWARNA), “Gemeinsame Informationsplattform Naturgefahren” [Joint Information Platform on Natural Hazards] (GIN)) or an amelioration of flood alarm systems (Bezzola et al. 2008; Bezzola and Hegg 2007, 2008). Analysis of the 2005 flood concluded that the intended direction in Swiss flood risk management proved of value, and the flood significantly impacted the shift toward an integrated risk management regime (Rohr et al. 2018). Therefore, we evaluated the 2005 flood as a focusing event.

In the aftermath of the 2005 flood, several smaller floods occurred. The flood in 2007 affected all regions of Switzerland and resulted in economic damage of CHF 375 million, while the floods in 2011, 2013, and 2014 were limited to certain regions and caused economic damage well below CHF 100 million. These floods further reinforced the integrated flood risk management approach in Switzerland by combining it with elements of other policy sectors’ actions, such as biodiversity (Biodiversity Strategy in 2012) or climate change adaptation (e.g., Climate Change Adaptation Strategy I in 2012 and II in 2014). However, these floods did not deploy focal power and cannot be classified as focusing events.

### Identifying drivers of change

To identify the drivers leading the three major flood events to deploy focal power and induce a paradigm shift, we compared their conditions to those of the other floods.

#### Floods’ objective magnitudes

We observed that floods’ objective magnitude only partially explained their identification as focusing events. First, considering the number of fatalities, we identified no clear pattern. The floods in 1868 (50 fatalities), 1910 (27 fatalities), and 2000 (16 fatalities) caused the highest number of flood victims, while the other floods showed low fatality numbers (between 0 and 9). As Pfister (2009) documented, the number of fatalities resulting from extreme events, including floods, has declined significantly since the nineteenth century. Consequentially, only the 1868 focusing event showed a high number of flood victims, while the two focusing events of 1987 and 2005 caused few flood victims. Therefore, the number of fatalities is not a key condition for identifying a focusing event.

Second, concerning the number of affected cantons, we could neither identify a clear pattern. Both focusing events in 1868 and 1987 each affected 5 cantons, the third focusing event in 2005 concerned 16 cantons. There were many floods with similar geographical outreach to the three focusing events, but also some floods with larger outreach, for instance, the floods in 1910 and 2007. We observed that some floods occurred in a few cantons only but affected a larger catchment area than other floods affecting numerous small cantons. Nevertheless, there was no indication that geographical outreach has an effect on the identification of a focusing event.

Third, we evaluated economic damage as having an impact on the identification of a focusing event. The three focusing events in 1868, 1987, and 2005 were those floods with the highest damage sum (CHF 1.4 billion, 1.3 billion, and CHF 2.8 billion, respectively). Despite a large range of measures adopted in the last century, more recent floods still caused significant damage, such as the floods in 1987 and 2005. According to Pfister (2009), extreme events’ damage sums have increased rapidly since 1977 due to a higher frequency of extreme events in the actual period, an expansion of settlement areas, and a higher value density near rivers. Thus, high economic damage seems to support floods’ identification as focusing events.

#### Media and parliamentary attention after floods

Figure 3a (in supplementary online information) illustrates the media coverage each year after a major flood event.^1^ Only the flood of 2005 received considerable media attention. All other floods, including the 1987 focusing event, did not receive significant public attention.

Similarly, parliamentary attention was also highest after the 2005 flood (Fig. 3b in the supplementary online information). Among the three focusing events,^2^ 2005 was the one with the highest coverage (16 cantons affected), which might explain the attention of the national parliament. For all other flood events, including the 1987 focusing event, there was no particular attention.

We needed to conclude at this stage that neither media nor parliamentary attention seemed a decisive factor turning a major flood into a focusing event. This was also confirmed by secondary literature, where we learned that, for example, in 1868, it was the Federal Council (government) and not the Parliament pushing for policy change (e.g., the 1874 constitutional infrastructure article and, subsequently, the 1976 Forest Inspectorate Act and the 1977 Hydraulic Engineering Inspectorate Act).

#### Subsystem properties absorbing floods

We observed that the presence of one or several coalitions and the activities of policy entrepreneurs matter when identifying a focusing event. Before the 1868 flood, the Swiss Forestry Association illustrated the link between population growth, deforestation, and increased flooding in the nineteenth century. However, the Forestry Association’s explanation had long been ignored but gained importance on the political agenda with the 1868 flood, and technical measures, such as river engineering and reforestation, were implemented (Pfister and Brändli 1999). In addition, the Forestry Association’s prominent representatives and direct connections to politics and science pushed their arguments. In contrast, despite diversified positions, hydraulic engineers marginally organized themselves and intervened moderately in the discussion on technical measures. Since forestry and hydraulic engineering pursued the same interests, the latter agreed with the Forestry Association to take the lead (Summermatter 2012). We concluded that a dominant *pro-infrastructure coalition* of forest and hydraulic engineering experts promoted the idea of technical flood prevention and pushed for a shift toward an infrastructural regime.

The second half of the twentieth century was shaped by the emerging environmental debate and the floods in 1978 and 1987. In three waves, a *pro-environment coalition* formed. First, around the 1950s, some actor groups called for nature and heritage protection through the limitations of hydropower, shipping, nuclear power, and other activities causing harm to nature (Summermatter 2012; Zaugg 2006). Second, around the 1970s, further actor groups promoted a comprehensive and holistic understanding of environmental protection and the renunciation of flood infrastructure. Third, around 1975, experts and the public administration initiated the design of flood risk management in a more environmentally friendly way, including spatial planning elements and other interests, such as nature protection and fishery (Summermatter 2012). These pro-environment developments were even strengthened by dynamics in the overarching water policy subsystem (Kissling-Näf and Kuks 2004; Varone et al. 2002): In 1957, the first water protection act entered into force and was strongly advocated by fishers as well as ecological associations. The second water protection act was then introduced in 1971 with mandatory sewage and a more holistic approach to water protection and natural flood risk management. However, strong opposition was noted from agriculture, municipalities, and landowners to natural flood risk management and also showed the trade-offs between public policies on one side and property and user rights on the other (Bolognesi & Nahrath 2020). As a result, two conflicting coalitions, the *pro-infrastructure coalition* and the *pro-environment coalition*, opposed each other. In the context of the 1987 flood and growing environmental protection and spatial planning legislation, the *pro-environment coalition* finally enforced its interests with numerous ecological aspects in the newly introduced Hydraulic Engineering Act of 1991 (Zaugg 2006).

Since the 1990s and in the context of the floods in 1993, 1999, and 2000, numerous actors have pushed for a shift toward a risk-based approach. In 2001, the Federal Office for Water and Geology, with the involvement of the three Federal Offices for Spatial Planning, for Environment, Forest, and Landscape, and for Agriculture, published a new guideline for flood risk management, prioritizing differentiated and integrated goals (Rohr et al. 2018). This path was reinforced by the flood in 2005, which resulted in a large number of developments that were strongly influenced by integrative, cross-sectoral, and multi-level elements, for instance, the expansion of weather forecasting, early warning systems, and emergency planning, as well as new communication strategies (Zischg et al. 2018). Thus, we observed that single federal agencies, with their particular interests and activities in the role of *policy entrepreneurs*, initiated an integration process and worked toward an integrated management of flood risks (Rohr et al. 2018).

## Discussion

Based on quantitative and qualitative data on major flood events and numerous flood policies since the mid-nineteenth century in Switzerland, we tested five hypotheses: whether an extreme event’s objective magnitude (H1), media and parliamentary attention (H2a and H2b), and subsystem dynamics, such as the presence of one or several coalitions (H3a) or the activities of policy brokers and entrepreneurs (H3b), influence if this extreme event can be identified as a focusing event and induces paradigm shift.

First, the magnitude of floods’ economic damage plays a decisive role in identifying a focusing event. The three identified focusing events in 1868, 1987, and 2005 show the largest damage sums of the 13 studied floods, and all induced paradigm shift. However, Hypothesis 1 could only partially be confirmed, as we included other indicators, such as fatalities or geographic scope for event magnitude, that seemed to play less of role, however. This speaks to the recent literature on how to conceive the magnitude of events and their role as triggers of change (Nohrstedt et al. 2021). And in sum, we can conclude that a major flood can be a necessary, but not a sufficient condition for change, as the subsequent discussion shows.

Second, media attention and parliamentary interventions following major flood events were not outstanding in their coverage, which is why we rejected hypotheses 2a and 2b. Only the 2005 flood event received both media and parliamentary attention; this might have been because its geographical coverage of 16 cantons was considerable. One possible explanation for the rather limited national attention, mainly in parliament, is that flood risk management is primarily a cantonal competence and of a regional extent. At the national level, flood risk management is limited to technocratic policymaking with the federal administration in charge (see Schulze et al. 2024 for diffusion across levels).

Third, the relevant subsystem’s dynamics matter when flood events occur. Over the period under investigation, first, a dominant pro-infrastructure coalition of forestry and hydraulic engineering experts became active, then the pro-infrastructure coalition competed with a new pro-environment coalition, and finally, four federal agencies acted as policy entrepreneurs and promoted policy change in their own but also in society’s interests. The focusing events in 1868, 1987, and 2005 thus opened a “window of opportunity” (Kingdon 1984) for a change of the status quo and provided opportunities for these change-prone actor groups to push forward new approaches. Or said differently, these actor groups were proved right by the flood events in their critique of the established regime. The three flood events could deploy their focusing power as they occurred at times of imminent change. They helped that long-developed underlying ideas got a maximum of attention and thus pushed the breakthrough of the emerging paradigm. Thus, we corroborated hypotheses 3a and 3b. From the test of these two hypotheses, we could confirm the following:

- A shock can induce policy learning of the dominant coalition (Sabatier 1998); this was the case in 1868, when the pro-infrastructure coalition’s requirements were boosted by the occurrence of the flood.
- Alternatively, a shock can affect the power balance, where the minority coalition benefits and may promote its ideas (Baumgartner and Jones 1993). The focusing event in 1987 confirmed the eroding interest in the long-dominant infrastructure policies that allowed the new pro-environment coalition to reframe flood risk management.
- Mainly the second and third paradigm shift were facilitated by the dynamics not only in the specific flood risk management subsystem, but also the overarching water policy subsystem: New laws, the strong presence of pro-environmental actors, and the trend for integration created some “positive spill-over” for paradigm change in flood risk management.
- A shock can push the particular interests of entrepreneurs, who bring innovation to the subsystem (Birkland 1997). After the 2005 flood, four federal agencies acted as entrepreneurs and advocated for a paradigm shift toward integrated risk management.

Relating these results back to our framework figure (Fig. 1), we conclude that besides a shock’s magnitude (e.g., economic damages), subsystem dynamics (e.g., coalitions and entrepreneurs) seem crucial to turn a shock into a focusing event and bring paradigm shift. Once an extreme event is absorbed by a change-prone environment, it can act as a catalyst and induce paradigm shift (Baumgartner and Jones 1993). However, it can take time until such an environment is created (Baumgartner 2013). This development is best illustrated by the second paradigm shift in Swiss flood risk management in the early 1990s. For decades, environmental organizations and citizen groups advocated more nature-oriented flood risk management (Zaugg 2006). However, the flood event in 1987 was the final trigger to deploy focal power and induce paradigm shift. So, the sequence as outlined in Table 1 is also confirmed: the subsystem must be “ready” for change when the event occurs; subsystem dynamics thus act as antecedent conditions to the (external) event. Subsequent political factors typically occurring after the event, such as public or parliamentary attention plaid less of a role. However, this holds true for Swiss flood risk management and might be different for other policy issues or countries.

## Conclusion

Some shocks — external or internal events to the political system — are identified as focusing events that induce major policy change (Birkland 1997), while the majority of them fade away without any political impact (Lindholm 2016). The aim of our article was to systematically study the conditions under which a shock can be identified as a focusing event, wields power to reframe current policies, and leads to a paradigm shift.

We studied two centuries of major flood events and paradigm shifts in Swiss flood risk management by applying the method of process tracing. The results illustrate that three flood events in 1868, 1987, and 2005 deployed focal power and each led to a paradigm shift. Two conditions made those floods turn into focusing events: the economic damage they induced and the presence of change-prone actors and coalitions in the respective policy subsystem (Fig. 1, Table 1). In addition to event characteristics, policymaking conditions are decisive in explaining when and why an event induces change (Birkland and Warnement 2016). So, the first conclusion is that major floods can be a necessary, but not sufficient condition to change. And the second conclusion is that the sequence of conditions matters: the change-prone environment seems an antecedent condition to the event. Slower developing subsystem dynamics and ideological trends must already be present when the event occurs (see Roland 2004). In turn, the event is then able to empower change-prone actors and create opportunity structures for coalitions and actors supporting innovation.

An interesting question for future research is whether, also in other countries or constituencies, the same two conditions (economic damage and the political situation in the respective policy subsystem) are relevant for turning a flood into a focusing event (see also Nohrstedt et al. 2021). Future research can also more systematically analyze the dynamics from other subsystems (e.g., outsider-insider dynamics). As we have shown, the flood risk management subsystem is embedded in the larger water policy subsystem (Varone et al. 2002) and the change-prone environment was surely also reinforced by the actors present in several water-related processes including flood management (see also Brandenberger et al. 2021). This also confirms the policy proximity assumption outlined by Nohrstedt and Weible (2010): if close or embedded subsystems are both or all ready for change, this might increase the chances for an event to deploy its focal power.

Finally, and apart from single case study analyses, such as ours, systematic comparative studies could be of interest, including, for instance, quantitative cross-field or cross-sector studies. Shocks seem to accompany policymaking for decades; however, they remain when we think about the nuclear accident of Fukushima, current, and future climate change, or the COVID-19 pandemic. Thus, it would be contextually relevant that policy scholars further develop their knowledge about the potential consequences of such shocks to make the political and policy subsystem more proactive than reactive, and thus prepared for them.

## Supplementary Information

Below is the link to the electronic supplementary material.

Supplementary file1 (DOCX 39 KB)

Supplementary file2 (PNG 65 KB)

Supplementary file3 (PNG 59 KB)

## Footnotes

1 Starting in 1910, as media data was not available beforehand. For more details, see also (Zemp 2015).

2 Note that for the flood in 1868 (and the ones in 1876 and 1910) no systematic data on parliamentary interventions was available yet in the consulted data base Curia Vista (Curia Vista 2021).
