# Supplementary material for: Under which conditions do extreme events support a paradigm shift? Studying focusing events during two centuries of Swiss flood risk management
Source: Reg Environ Change. 2024 Oct 23;24(4):162. doi: 10.1007/s10113-024-02316-2 (PMC11496349; doi:10.1007/s10113-024-02316-2)
Supplement: Supplementary file 1 — Supplementary file1 (DOCX 39 KB) [file 10113_2024_2316_MOESM1_ESM.docx]

**Supplementary Online Material**

Table 2 shows a systematic review of all regulations related to flood risk management between 1848 and 2020 in Switzerland, i.e., constitutional articles, acts, ordinances, strategies, and platforms. These regulations have been considered to identify flood regimes and paradigm shifts.

Table 2 List of regulations to identify flood regimes and paradigm shifts

| **Decision** | **In force** | **Regulation** | **SR**^a^ | **Type** | **Amendment** |
| --- | --- | --- | --- | --- | --- |
| 12.09.1848 | 12.09.1848 | Federal Constitution; Article 21 on Infrastructure | 101 | Constitution | Introduction |
| 29.05.1874 | 29.05.1874 | Federal Constitution; Article 24 on Hydraulic Engineering Inspectorate and Forest Inspectorate | 101 | Constitution | Revision |
| 24.03.1876 | 24.03.1876 | Forest Inspectorate Act | 921.0 | Act | Introduction |
| 22.06.1877 | 06.10.1877 | Hydraulic Engineering Inspectorate Act | 721.10 | Act | Introduction |
| 11.10.1902 | 01.04.1903 | Forest Inspectorate Act | 921.0 | Act | Revision |
| 22.12.1908 | 22.12.1908 | Federal Constitution; Article 24^bis^ on Hydroelectrical production | 101 | Constitution | Revision |
| 22.12.1916 | 01.01.1918 | Water Rights Act | 721.80 | Act | Introduction |
| 30.09.1953 | 25.03.1954 | Federal Constitution; Article 24^quater^ on Water Protection | 101 | Constitution | Revision |
| 16.03.1955 | 01.01.1957 | Water Protection Act | 814.21 | Act | Introduction |
| 21.03.1969 | 11.12.1969 | Federal Constitution; Article 22^ter and quater^ on Spatial Planning | 101 | Constitution | Revision |
| 24.06.1971 | 01.01.1972 | Federal Constitution; Article 24^septies^ on Environmental Protection | 101 | Constitution | Revision |
| 08.10.1971 | 01.07.1972 | Water Protection Act | 814.20 | Act | Revision |
| 20.06.1975 | 07.12.1975 | Federal Constitution; Article 24^bis and quater^ on Water Management | 101 | Constitution | Revision |
| 22.06.1979 | 01.01.1980 | Spatial Planning Act | 700 | Act | Introduction |
| 1982 | 1982 | “Hochwasserschutz an Fliessgewässern” [*Flood control at rivers and streams* ] |  | Guideline | Introduction |
| 07.10.1983 | 01.01.1985 | Environmental Protection Act | 814.01 | Act | Introduction |
| 24.01.1991 | 01.11.1992 | Water Protection Act | 814.20 | Act | Revision |
| 21.06.1991 | 01.01.1993 | Hydraulic Engineering Act | 721.100 | Act | Introduction |
| 04.10.1991 | 01.01.1993 | Forest Act | 921.0 | Act | Introduction |
| 1997 | 1997 | National Platform for Natural Hazards PLANAT |  | Platform | Establishment |
| 2000 | 2003 | “Sicherheit vor Naturgefahren – Vision und Strategie” [*Protection against natural hazards – vision and strategy*] |  | Strategy PLANAT | Introduction |
| 2001 | 2001 | “Hochwasserschutz an Fliessgewässern” [*Flood control at rivers and streams*] |  | Guideline | Introduction |
| 2003 | 2005 | *“*Strategie Naturgefahren Schweiz – Synthesebericht” *[Strategy Natural Hazards Switzerland – Synthesis Report*] |  | Strategy PLANAT | Introduction |
| 2005 | 2009 | Implementation of Natural Hazards Action Plan I |  | Strategy PLANAT | Introduction |
| 2009 | 2011 | Implementation of Natural Hazards Action Plan II |  | Strategy PLANAT | Introduction |
| 18.08.2010 | 01.01.2011 | Alerting and Security Radio Ordinance | 520.12 | Ordinance | Introduction |
| 2010 | 2010 | *“*Optimierung der Warnung und Alarmierung” [*Optimization of warning and alerting*] (OWARNA) |  | Platform | Establishment |
| 2010 | 2010 | *“*Gemeinsame Informationsplattform Naturgefahren” [Joint Information Platform on Natural Hazards] (GIN) |  | Platform | Establishment |
| 2012 | 2012 | “Anpassung an den Klimawandel in der Schweiz - Erster Teil der Strategie des Bundesrates vom 2. März 2012” [Adaptation to climate change in Switzerland – First part of the Federal Council’s strategy. Adopted on 2 March 2012] |  | Strategy | Introduction |
| 2012 | 2012 | “Strategie Biodiversität Schweiz” [*Swiss Biodiversity Strategy*] |  | Strategy | Introduction |
| 2014 | 2014 | “Aktionsplan 2014–2019. Zweiter Teil der Strategie des Bundesrates vom 9. April 2014” [Action Plan 2014-2019. Second Part of the Federal Council’s Strategy of 9 April 2014] |  | Strategy | Introduction |
| 2016 | 2016 | “Strategie Nachhaltige Entwicklung 2016-2019” [*Sustainable Development Strategy 2016-2019*] |  | Strategy | Introduction |
| 2018 | 2018 | “Strategie 2018 – Umgang mit Risiken aus Naturgefahren” [2018 strategy Management of risks from natural hazards] |  | Strategy PLANAT | Introduction |

^a^ Number in the classified compilation of Swiss federal law (“Systematische Sammlung des Bundesrechts”)

Source: Leimbacher and Perler (2000), Mauch, Reynard, and Thorens (2000), and Zaugg Stern (2006).

Table 3 shows a systematic review of all parliamentary interventions submitted during the first or second parliamentary session following each major flood event. These parliamentary interventions have been considered to analyze the condition of ‘parliamentary attention’.

Table 3 List of parliamentary interventions to identify focusing events

| **Year flood** | **Date flood** | **Date intervention** | **Date response** | **No., name, title** | **Kind of intervention** |
| --- | --- | --- | --- | --- | --- |
| 1868 | 27.09.-04.10. | - | - | - | - |
| 1876 | 10.-15.06. | - | - | - | - |
| 1910 | 14.–15.06. | - | - | - | - |
| 1978 | 06.-07.08. | 18.09.1978 | - | 78.460 Barchi – Überschwemmungen im Kanton Tessin und im Misox | Parlamentarische Initiative |
|  |  | 18.09.1978 | - | 78.463 Pedrazzini – Erdrutsch im Campo Vallemaggia | Interpellation |
| 1987 | 18.07.;  24.–25.08. | 21.09.1987 | 25.11.1987 | 87.539 Günter – Unwetterschäden und Umweltbelastung | Postulat |
|  |  | 21.09.1987 | 25.11.1987 | 87.540 CVP-Fraktion – Unwetterschäden. Langfristige Vorbeugungsmassnahmen | Motion |
|  |  | 21.09.1987 | 25.11.1987 | 87.545 SP-Fraktion – Unwetterkatastrophen. Analyze und Vorbeuge Massnahmen | Interpellation |
|  |  | 28.09.1987 | 28.09.1987 | 87.5090 Fankhauser – Probealarm. Meldungen am Radio | Frage |
|  |  | 09.10.1987 | 25.11.1987 | 87.906 Grendelmeier – Evakuationspläne | Parlamentarische Initiative |
| 1993 | 24.09.;  12.-13.10. | 27.09.1993 | 04.10.1993 | 93.1061 Schmidhalter – Unwetterschäden September 1993 | Dringliche einfache Anfrage |
|  |  | 28.09.1993 | 07.10.1993 | 93.3424 Bloetzer – Unwetterkatastrophe im Wallis | Interpellation |
|  |  | 30.09.1993 | 17.11.1993 | 93.3438 SP-Fraktion – Unwetterschäden in der Schweiz | Interpellation |
|  |  | 04.10.1993 | 04.10.1993 | 93.5152 Comby – Katastrophe von Brig | Frage |
|  |  | 04.10.1993 | 04.10.1993 | 93.5169 Jenni – Hilfe für die Region Brig | Frage |
|  |  | 04.10.1993 | 04.10.1993 | 93.5174 Hildbrand – Unwetterschäden am Simplon. Wiederaufnahme des Autoverlads | Frage |
|  |  | 06.10.1993 | Zurückgezogen | 93.3482 Bloetzer – Umfassende Abklärung der Geschehnisse anlässlich der Unwetterschäden im Saastal | Interpellation |
|  |  | 02.12.1993 | 23.03.1994 | 93.1094 Cavadini – Unwetterschäden im Tessin. Unterstützung des Bundes | Dringliche einfache Anfrage |
| 1999 | 11.–15.05.;  20.-25.05. | 01.06.1999 | 23.06.1999 | 99.1070 Stucky – Vorsorgliche Massnahmen gegen Überschwemmungen | Dringliche Interpellation |
|  |  | 17.06.1999 | 08.09.1999 | 99.3315 Delalay – Für eine effiziente Vorbeugung bei Katastrophen | Empfehlung |
|  |  | 18.06.1999 | 25.08.1999 | 99.3364 Raggenbass – Bodenseeregulierung | Postulat |
|  |  | 30.08.1999 | 27.09.1999 | 99.1114 Schmid – Lawinenschäden 1999. Subventionierung Durch den Bund | Dringliche einfache Anfrage |
|  |  | 31.08.1999 | 17.11.1999 | 99.3407 David – Linthkanal. Hochwasserschutz und ökologische Aufwertung | Postulat |
|  |  | 27.09.1999 | 27.09.1999 | 99.5111 Epiney – Von Lawinen und Unwettern betroffene Gemeinwesen. Bankkredite | Frage |
|  |  | 27.09.1999 | 27.09.1999 | 99.5122 Debons – Kredite betreffend Unwetterschäden | Frage |
|  |  | 06.10.1999 | 13.12.1999 | 99.1153 Bloetzer – Stärkung der Abwehr von Naturgefahren | Einfache Anfrage |
| 2000 | 14.-15.10. | 04.12.2000 | 14.02.2001 | 00.3634 Abate – Locarno wie Venedig | Interpellation |
|  |  | 14.12.2000 | 14.02.2001 | 00.3699 Eymann – Überschwemmungen im Tessin. Massnahmen zur Verhinderung | Postulat |
|  |  | 15.12.2000 | 28.02.2001 | 00.3750 Günter – Projekt Waldgrenze | Postulat |
|  |  | 07.03.2001 | 03.07.2001 | 01.3045 Wyss – Ende des Permafrosts. Folgen für die Schweizer Alpen | Interpellation |
| 2005 | 20.–23.08. | 12.09.2005 | 29.03.2006 | 05.3478 UREK-N – Unwetterkatastrophe 2005 | Interpellation |
|  |  | 20.09.2005 | 30.09.2005 | 05.3472 UREK-S – Unwetterkatastrophe 2005 | Dringliche Interpellation |
|  |  | 21.09.2005 | 01.02.2006 | 05.3485 Grüne Fraktion – Unwetter. Dringliche Massnahmen sind notwendig | Interpellation |
|  |  | 21.09.2005 | 01.02.2006 | 05.3486 SP-Fraktion – Hochwasser in der Schweiz. Präventionsmassnahmen | Interpellation |
|  |  | 03.10.2005 | 03.10.2005 | 05.5191 Wyss – Unwetter 2005. Folgen verheerender Sparpolitik? | Frage |
|  |  | 03.10.2005 | 03.10.2005 | 05.5192 Wyss – Unwetter 2005. Politische Fahrlässigkeit? | Frage |
|  |  | 03.10.2005 | 03.10.2005 | 05.5199 Lang – Unwetterkatastrophen | Frage |
|  |  | 03.10.2005 | 03.10.2005 | 05.5200 Graf – Unwetterkatastrophen | Frage |
|  |  | 03.10.2005 | 03.10.2005 | 05.5201 Hollenstein – Unwetterkatastrophen | Frage |
|  |  | 03.10.2005 | 03.10.2005 | 05.5202 Vischer – Unwetterkatastrophen | Frage |
|  |  | 03.10.2005 | 03.10.2005 | 05.5204 Menétrey-Savary – Unwetterkatastrophen | Frage |
|  |  | 03.10.2005 | 03.10.2005 | 05.5207 Menétrey-Savary – Unwetter 2005. Dringliche Massnahmen? | Frage |

| **Year flood** | **Date flood** | **Date intervention** | **Date response** | **No., name, title** | **Kind of intervention** |
| --- | --- | --- | --- | --- | --- |
|  |  | 03.10.2005 | 03.10.2005 | 05.5208 Genner – Unwetterkatastrophen | Frage |
|  |  | 03.10.2005 | 03.10.2005 | 05.5209 Bühlmann – Unwetterkatastrophen | Frage |
|  |  | 03.10.2005 | 03.10.2005 | 05.5210 Marty Kälin – Prävention und Hochwasser | Frage |
|  |  | 03.10.2005 | 03.10.2005 | 05.5211 Lustenberger – Unwetterschäden 2005 | Frage |
|  |  | 03.10.2005 | 03.10.2005 | 05.5214 Leuenberger – Dringliche Massnahmen gegen Überschwemmungen und Lawinen? | Frage |
|  |  | 03.10.2005 | 03.10.2005 | 05.5215 Leuenberger – Gefahrenkarten rasch erstellen | Frage |
|  |  | 03.10.2005 | 03.10.2005 | 05.5225 Allemann – Wirksamer Hochwasserschutz | Frage |
|  |  | 06.10.2005 | 01.02.2006 | 05.3580 Schenk – Waldbewirtschaftung und Hochwasserprävention. Massnahmen | Interpellation |
|  |  | 06.10.2005 | 01.02.2006 | 05.3586 Grüne Fraktion – Naturgefahren vermindern und verhindern | Motion |
|  |  | 06.10.2005 | 01.02.2006 | 05.3629 Teuscher – Mehr Raum für Fliessgewässer | Motion |
|  |  | 06.10.2005 | 01.02.2006 | 05.3630 Teuscher – Genügend Mittel für Hochwasserschutz | Motion |
|  |  | 07.10.2005 | 15.02.2006 | 05.3661 Leutenegger Oberholzer – Schweiz 2005. Naturgefahren. Folgerungen | Interpellation |
|  |  | 07.10.2005 | 01.02.2006 | 05.3664 Allemann – Hochwasserschutz. Mehr Raum für Flüsse und Bäche | Motion |
|  |  | 07.10.2005 | 01.02.2006 | 05.3673 Marty Kälin – Zusammenhang zwischen Bodenversiegelung und Unwetterschäden | Postulat |
|  |  | 07.10.2005 | 01.02.2006 | 05.3674 Marty Kälin – Zeitpunkt und Zuständigkeit bei einer Unwetterwarnung | Interpellation |
|  |  | 07.10.2005 | 29.03.2006 | 05.3689 Wyss – Hochwasserkatastrophe. Folge der Sparmassnahmen | Motion |
|  |  | 07.10.2005 | 23.11.2005 | 05.3692 Wyss – Einheitliches meteorologisches Warnsystem | Motion |
|  |  | 15.12.2005 | 22.02.2006 | 05.3839 Büchler – Hochwasserschutz beim Überlastfall | Interpellation |
| 2007 | 08.–09.08. | 17.09.2007 | 07.11.2007 | 07.3566 Reimann – Hochwasser-Abfluss am Bielersee. Überschwemmungskatastrophe in aareabwärts gelegenen Regionen | Interpellation |
|  |  | 26.09.2007 | 28.11.2007 | 07.3590 Stadler – Konzept zur Gewährleistung der langfristigen Sicherheit vor Naturgefahren | Motion |
|  |  | 01.10.2007 | 01.10.2007 | 07.5322 Simoneschi-Cortesi – Klimawandel. Karte der Gefahrengebiete und Planung von Massnahmen | Frage |
|  |  | 03.10.2007 | 07.03.2008 | 07.1100 Lang – Klima, Wasser und Entwicklung | Anfrage |
|  |  | 03.10.2007 | 20.02.2008 | 07.3625 Cathomas – Kampf gegen Naturgefahren | Motion |
|  |  | 04.10.2007 | Zurückgezogen | 07.3643 Mathys – Aare-Abfluss am Bielersee. Hochwasser im Aargau | Interpellation |
|  |  | 21.12.2007 | 25.09.2008 | 07.499 Zisyadis – Aufnahme des Vorsorgegrundsatzes in die Bundesverfassung | Parlamentarische Initiative |
| 2011 | 10.–11.10. | 15.03.2012 | 16.05.2012 | 12.3176 Wandfluh – Wirkungsvoller Hochwasserschutz. Unterhalt entlang von Fliessgewässern in Schutzgebieten und Rodung der Ufervegetation | Motion |
| 2013 | 01.-02.05.;  31.05.–02.06. | 27.09.2013 | 20.11.2013 | 13.3933 Rytz – Schaffung eines Klima-Ausgleichsfonds für die alpinen Regionen | Postulat |
|  |  | 13.12.2013 | 19.02.2014 | 13.4267 Amherd – Masterplan Wasser. Erarbeitung von Grundlagen zur langfristigen wasserwirtschaftlichen Strategie in den Gebirgsregionen | Motion |
| 2014 | 22.-24.07.;  10.-11.08. | 26.09.2014 | 05.11.2014 | 14.3941 Schibli – Fallholz entlang von Gewässern wegräumen | Motion |
|  |  | 12.12.2014 | 25.02.2015 | 14.4289 Fischer – Quantifizierung und Differenzierung der Naturgefahrenrisiken für Nationalstrasse und Eisenbahn am Axen | Interpellation |

Source: Swiss parliamentary data base Curia Vista (Curia Vista, 2021).

**Fig. 3a** Media coverage one year after each flood event for seven out of the twelve floods, and three out of four focusing events (for details, see Zemp, 2015)

**Fig. 3b** Parliamentary interventions (including all different types, e.g., motions, postulates, interpellations, etc.) during two parliamentary sessions following each major flood event (time period of 4-6 months)
