# Supplementary figures and images for: Under which conditions do extreme events support a paradigm shift? Studying focusing events during two centuries of Swiss flood risk management
Source: Reg Environ Change. 2024 Oct 23;24(4):162. doi: 10.1007/s10113-024-02316-2 (PMC11496349; doi:10.1007/s10113-024-02316-2)

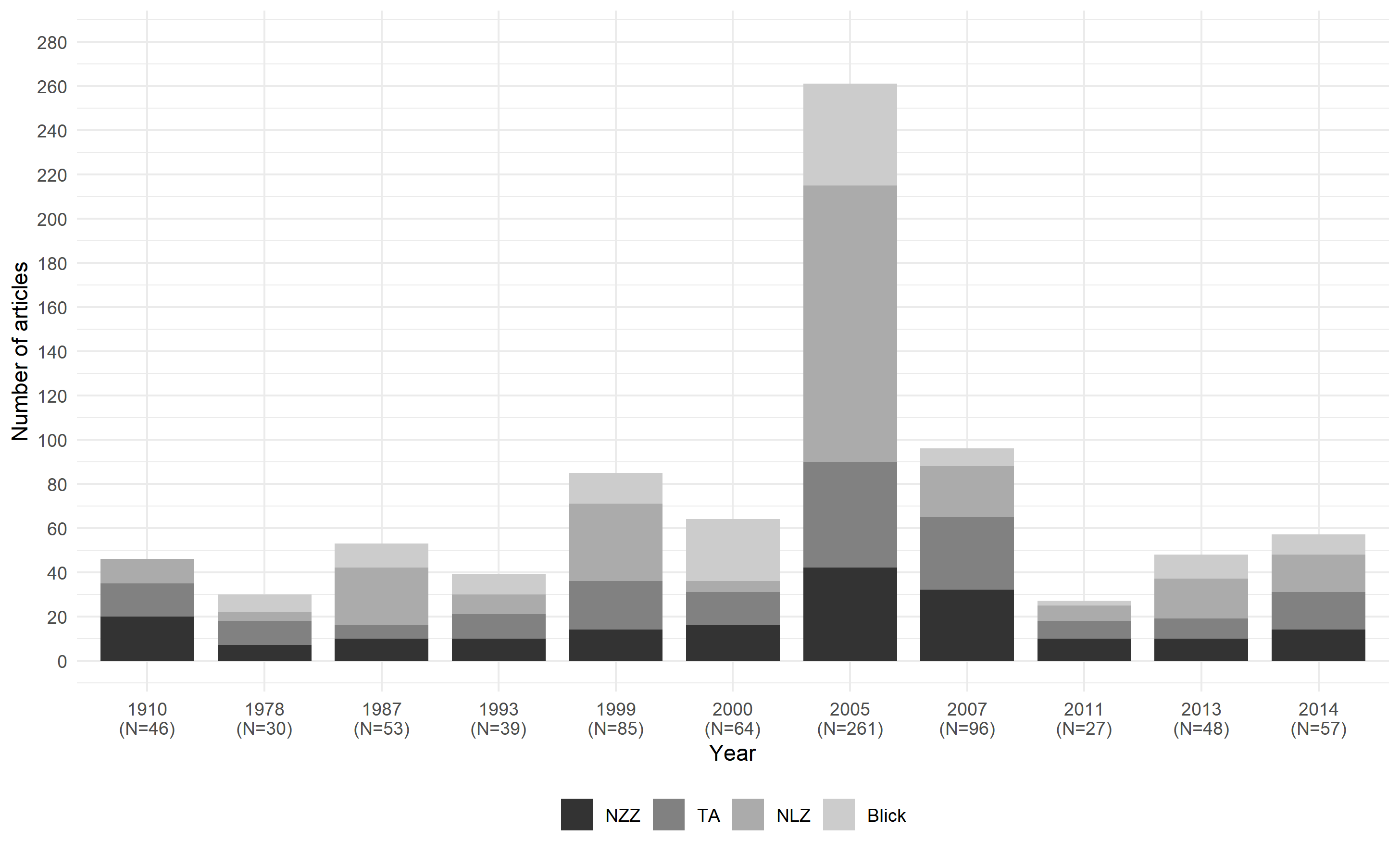

Supplement: Supplementary file 2 — Supplementary file2 (PNG 65 KB) [file 10113_2024_2316_MOESM2_ESM.png]

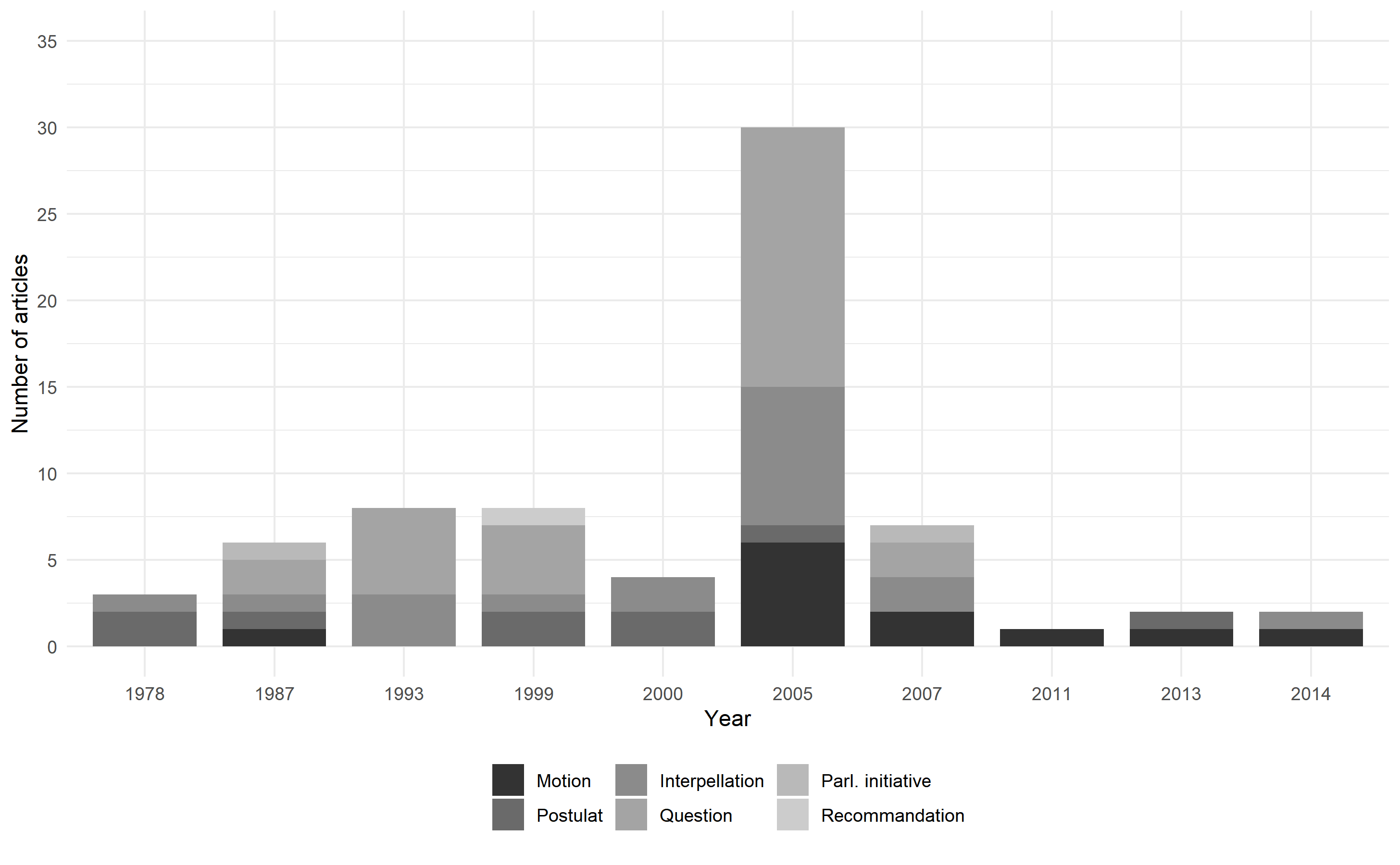

Supplement: Supplementary file 3 — Supplementary file3 (PNG 59 KB) [file 10113_2024_2316_MOESM3_ESM.png]
